# Supplementary material for: DNA-Templated Fluorescent Silver Nanoclusters Inhibit Bacterial Growth While Being Non-Toxic to Mammalian Cells
Source: Molecules. 2021 Jul 1;26(13):4045. doi: 10.3390/molecules26134045 (PMC8271471; doi:10.3390/molecules26134045)
Supplement: Supplementary file 1 [file molecules-26-04045-s001.zip › molecules-1200580-supplementary.pdf]

SUPPORTING INFORMATION

DNA-templated Fluorescent Silver Nanoclusters Inhibit Bacterial Growth while being Non-toxic to Mammalian Cells

Lewis Rolband[1#], Liam Yourston[2#], Morgan Chandler[#1], Damian Beasock[#1], Leyla Danai[1], Seraphim Kozlov[1], Nolan Marshall[2], Oleg Shevchenko[1], Alexey V. Krasnoslobodtsev[2]*, and Kirill A. Afonin[1]*

[1]Nanoscale Science Program, Department of Chemistry, University of North Carolina at Charlotte, Charlotte, NC 28223, USA
[2]Department of Physics, University of Nebraska at Omaha, Omaha, NE 68182, USA

SEQUENCES USED IN THIS PROJECT

All oligonucleotides were purchased from IDT, Inc. Names denote the numbers of cytosines in each hairpin loop, underlined below.[1,2]

C7: 5'- TATCCGTCCCCCCCACGGATA
C9: 5'- TATCCGTCCCCCCCCCACGGATA
C11: 5'- TATCCGTCCCCCCCCCCCACGGATA
C13: 5'- TATCCGTCCCCCCCCCCCCCACGGATA

From Fig. S1:
C12: 5'- TATCCGTCCCCCCCCCCCCACGGATA
Single-stranded C12: 5'- CCCCCCCCCCCC

SUPPORTING METHODS

Bacterial growth of* Lactobacilli *on LB agar. One colony with the morphology of *Lactobacilli* was isolated from 1:100 and 1:1000 dilutions of Bulgarian yogurt (White Mountain) tested on LB agar plates in duplicate and was inoculated into SOS medium (Invitrogen) to prepare a pure culture. The culture was incubated at 42-45 °C for 36 hours to produce a turbid culture. A 100 µL aliquot of this culture was transferred into fresh SOS medium and again incubated at 42-45 °C for 36 hours to adapt *Lactobacilli* to SOS medium. This culture was then used for all subsequent experiments and was stored at 4 °C between experiments to prevent bacterial cells from proliferating. Each DNA-templated AgNC (C7, and C9) was mixed with liquid *Lactobacilli* culture in SOS media so that the final dilutions of bacteria were 1:1000 and 1:10,000 with nanoclusters at the final concentration of 22.5 µM of silver in 100 µL. From this, 25 µL of each sample was plated per one quarter of an LB agar plate; two replicates per sample were tested. Plates were incubated at 42-45 °C for 36 hours, then individual colonies were counted.

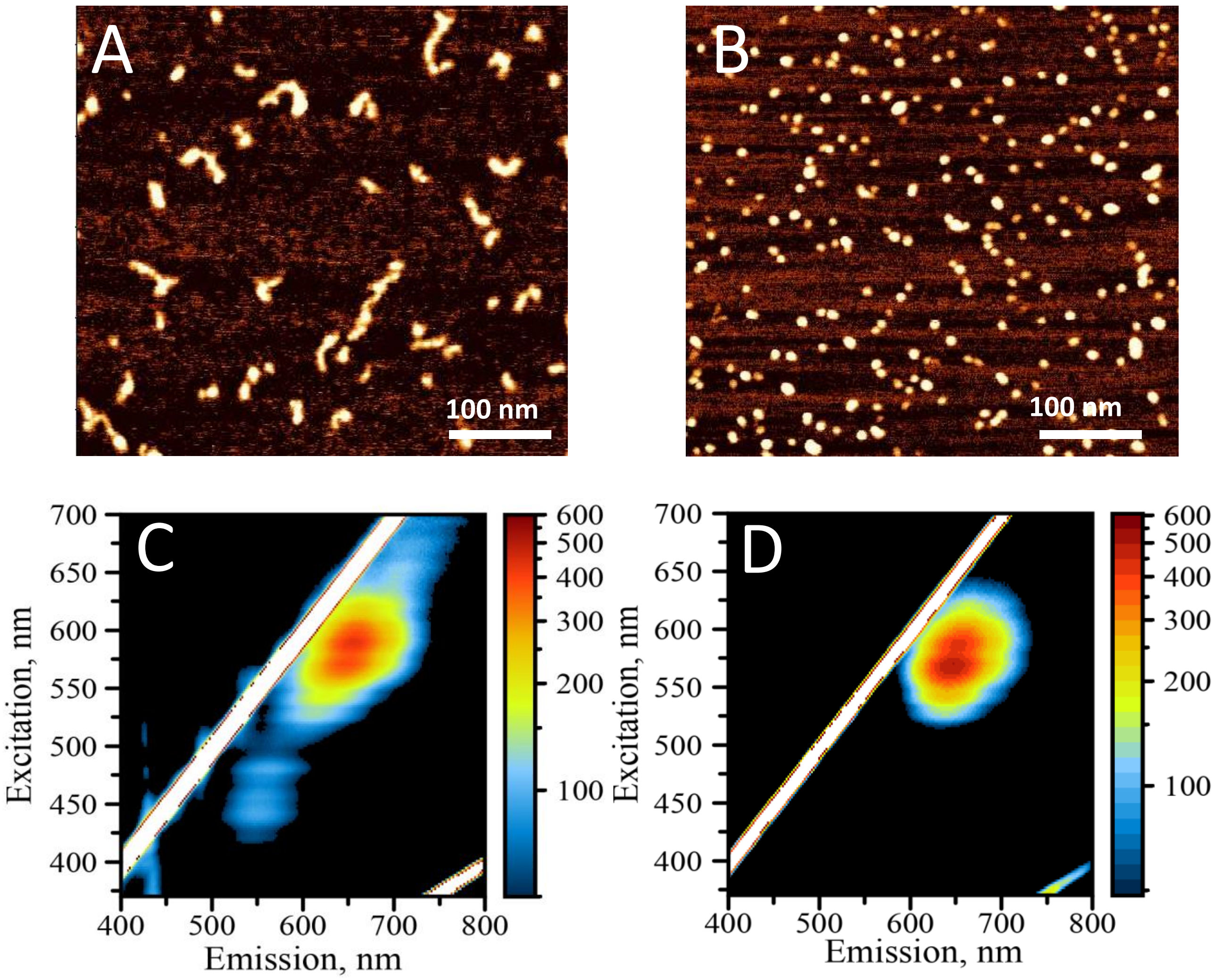

SI Figure S1. Comparison of C12 template as a single-stranded nucleotide or as a loop in a hairpin structure. **(A)** AFM image of AgNCs formed on a template containing single-stranded C12 sequence, **(B)** AFM image of AgNCs formed on a template containing C12 loop, **(C)** EEM of AgNCs templated on a template containing single-stranded C12 sequence, **(D)** EEM of AgNCs templated on a template containing C12 loop.

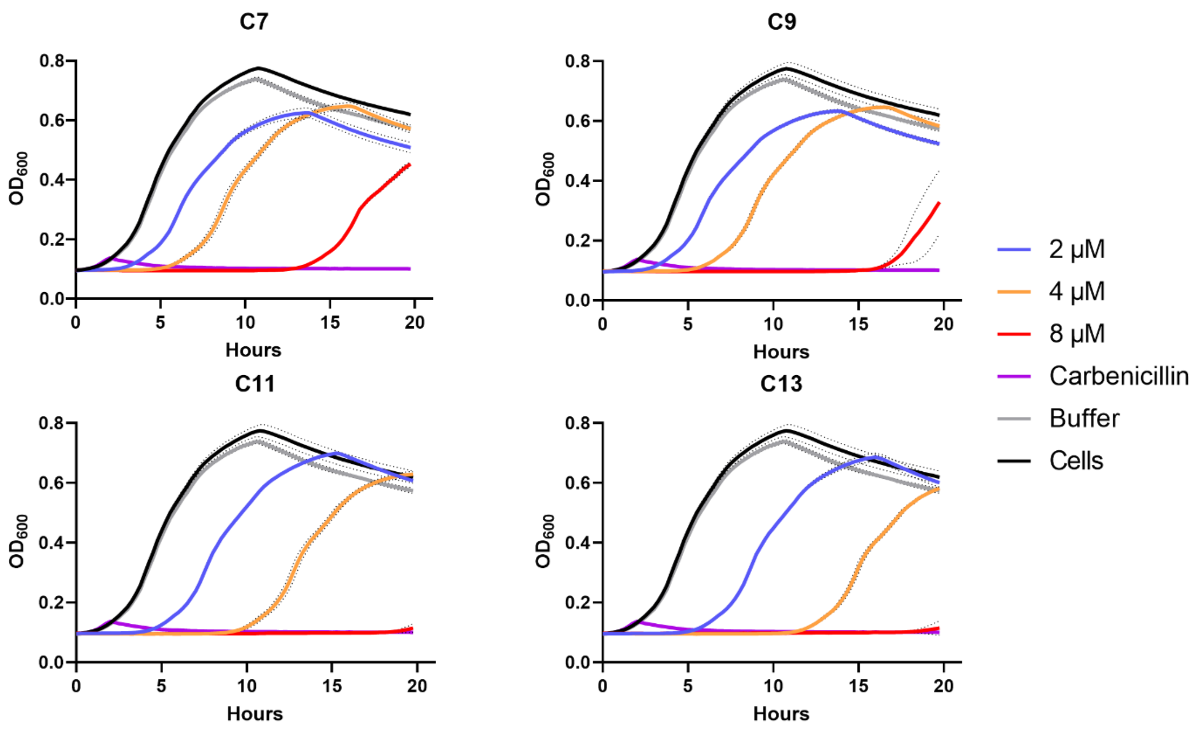

SI Figure S2. Liquid culture growth curves are shown of *E. coli* which have been treated with varying concentrations of AgNC. Each is shown with the standard deviation as a dotted line on either side of the solid line in the same color. There is a strong dose-dependence for each AgNC with 8 μM C11 eliminating growth for almost the full 20 hours.

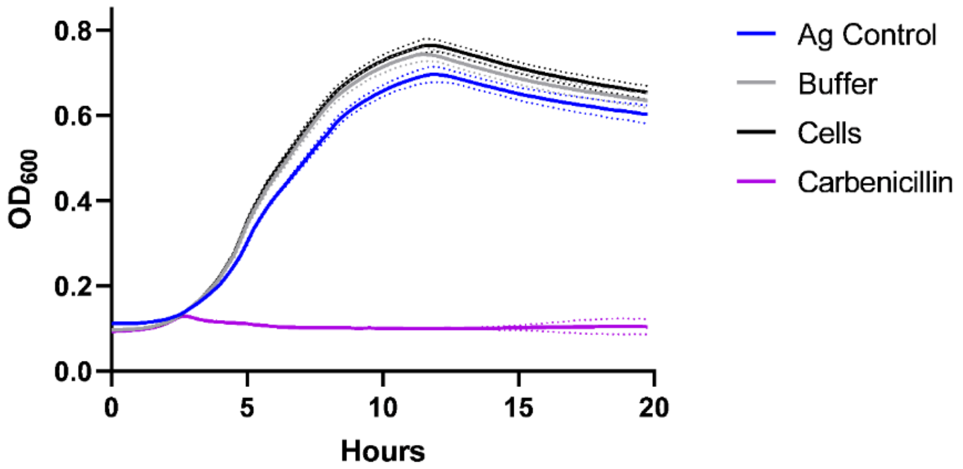

SI Figure S3. To see the effects of free silver that may not have bound to DNA, 650 μM $AgNO_3$ solution was reduced with an equimolar amount of $NaBH_4$, just as in the synthesis of the C13 AgNC. *E. coli* was then treated with the same amount of this solution as would be present in 4 μM C13 AgNC treatments. While there was a slight reduction in growth rate and the maximum OD_{600}, these were minimal compared to the effects of the C13 AgNC at 4 μM.

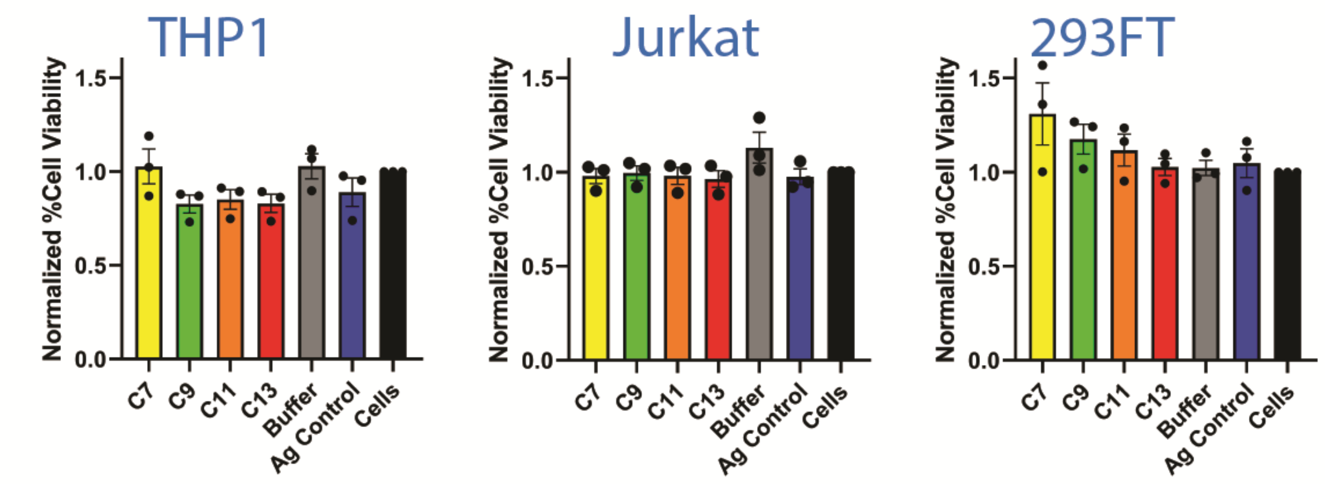

SI Figure S4. Additional mammalian cell viability assays were conducted with 8 µM AgNC concentrations following the methods described in the main text. Following 20 hours of incubation at 37 °C and 5% CO_2, MTS was added and incubated for an additional 75 minutes at the same conditions. The absorbance was recorded at 490 nm and the relative cell viability was calculated. No statistically significant reduction in cell viability was found at the elevated AgNC conditions.

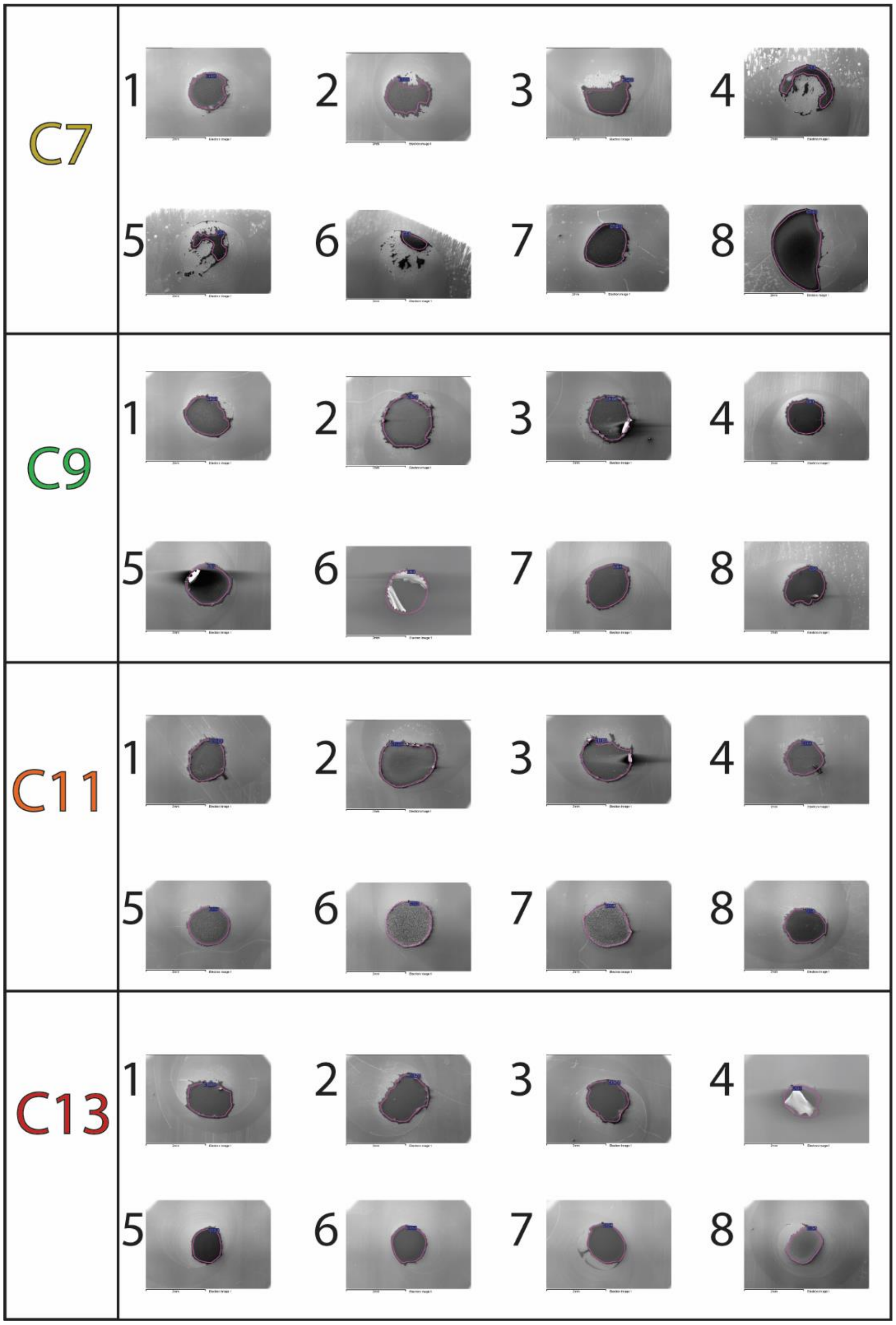

SI Figure S5. Secondary electron micrographs of all of the dried AgNC samples which were used for the stoichiometry calculations are shown.

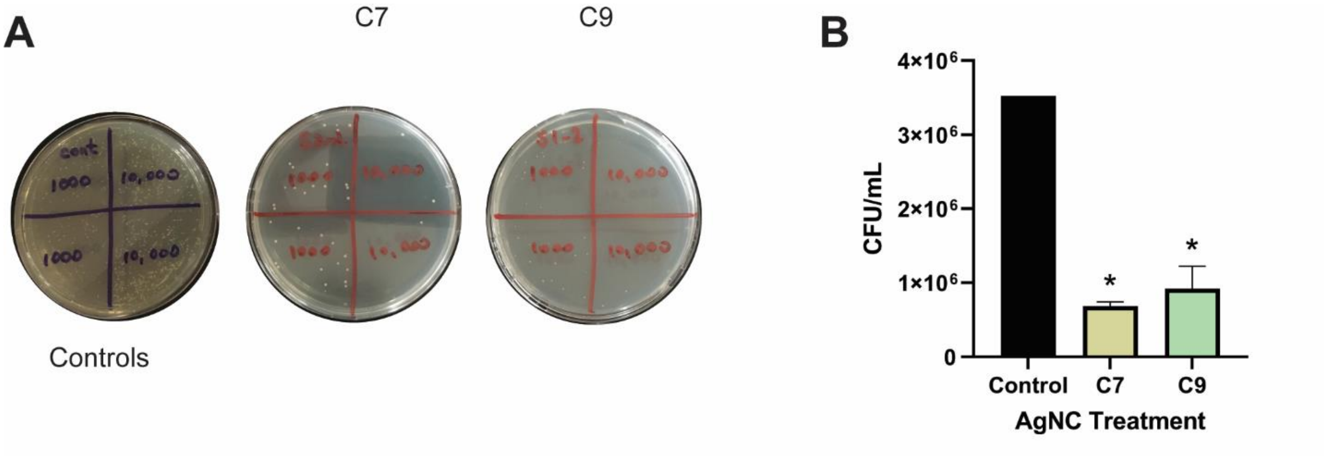

SI Figure S6. (A) Plates of *Lactobacilli* treated with C7 or C9 AgNCs and the resulting colonies formed. **(B)** Colony forming units (CFU)/mL after treatment with C7 or C9 were compared to the control sample Significance of $p<0.05$ is denoted with an asterisk.

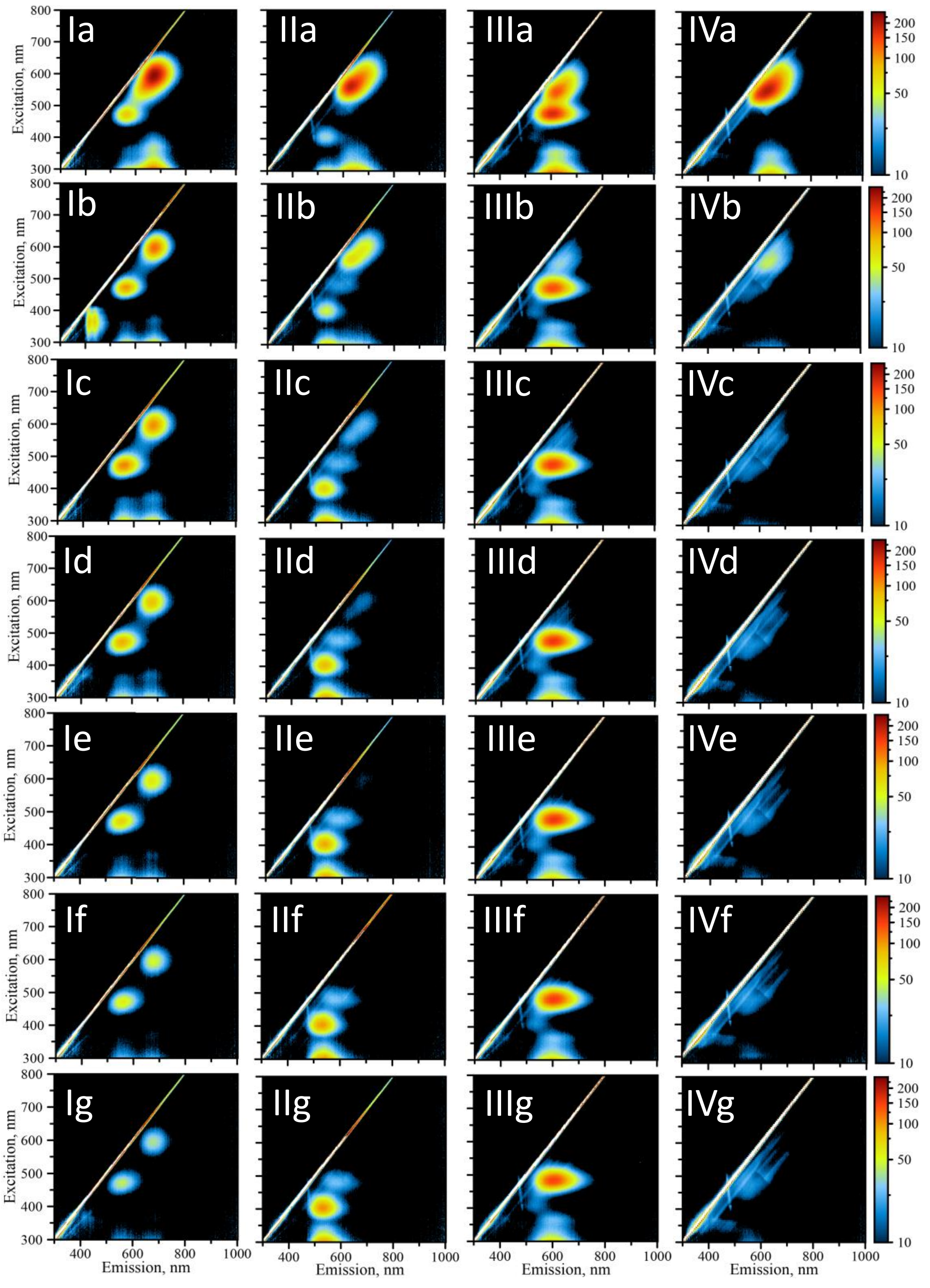

SI Figure S7. Titration of AgNCs with hydrogen peroxide. I) C7 DNA-AgNCs, II) C9 DNA-AgNCs, III) C11 DNA-AgNCs, IV) C13 DNA-AgNCs. Progressive addition of hydrogen peroxide shows changes in oxidative state of DNA-AgNCs. (**a**-**f**) are different ratios of C_{AgNC}/C_{H2O2} = 1/0 (**a**), 1/1.9 (**b**), 1/3.8 (**c**), 1/5.7 (**d**), 1/7.6 (**e**), 1/9.5 (**f**), 1/11.4 (g).

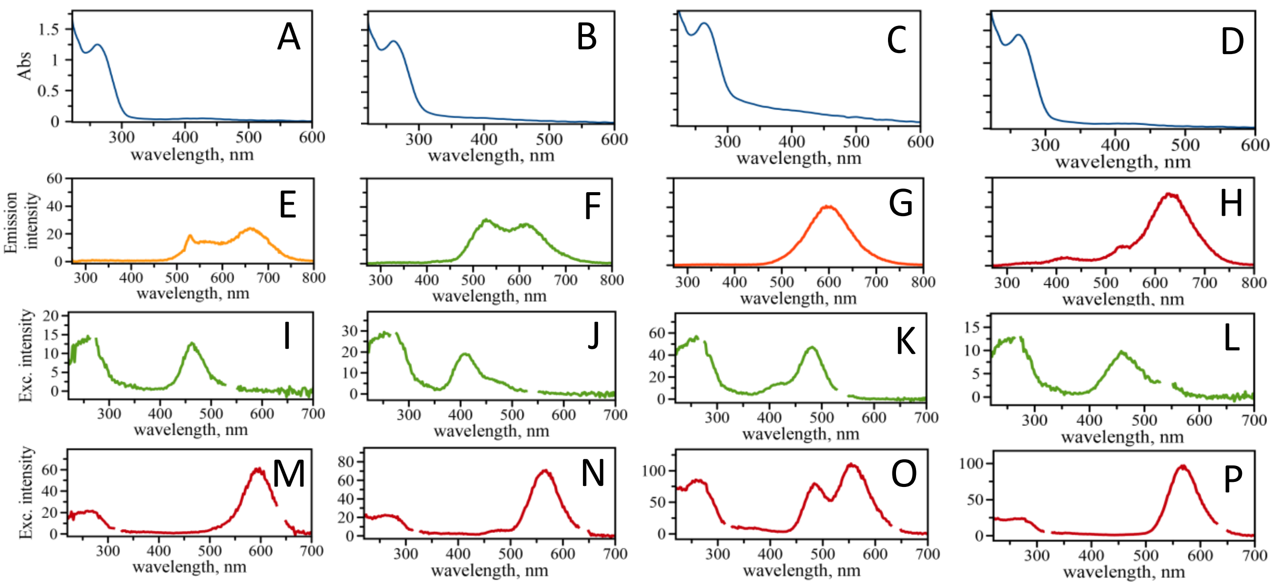

SI Figure S8. Evaluation of optical properties. UV-Vis spectra of A) C7, B) C9, C) C11, D) C13 samples immediately after purification. Emission spectra with 254 nm excitation mimicking color observation shown in Figure 1, E) C7, F) C9, G) C11, H) C13. Excitation spectra for 525 nm emission peak, I) C7, J) C9, K) C11, L) C13. Excitation spectra for 635 nm emission peak, M) C7, N) C9, O) C11, P) C13.

SI Table S1. The calculated number of silver atoms from the atomic percentages obtained from the EDS reports are shown with the average and standard error of the mean for each AgNC.

	1	2	3	4	5	6	7	8	Mean		SEM
**C7**	11.3	11.5	9.7	12.3	8.3	9.2	8.0	8.7	9.9	±	0.6
**C9**	10.2	7.9	11.4	7.4	7.8	7.6	8.0	7.7	8.5	±	0.5
**C11**	11.4	9.8	14.0	11.2	13.6	10.8	11.8	11.4	11.7	±	0.5
**C13**	11.3	13.3	10.9	8.0	7.5	7.9	8.7	13.7	10.2	±	0.9

Supporting References

(1) O'Neill, P. R.; Gwinn, E. G.; Fygenson, D. K. UV Excitation of DNA Stabilized Ag Cluster Fluorescence via the DNA Bases. *The Journal of Physical Chemistry C* **2011**, *115*, 24061-24066.

(2) O'Neill, P. R.; Velazquez, L. R.; Dunn, D. G.; Gwinn, E. G.; Fygenson, D. K. Hairpins with Poly-C Loops Stabilize Four Types of Fluorescent Agn:DNA. *The Journal of Physical Chemistry C* **2009**, *113*, 4229-4233.
